# Epigenetic aging biomarkers in dietary geroscience: feasibility, participant perceptions, and trial design considerations

**DOI:** 10.1007/s11357-026-02443-0

**Published:** 2026-07-29

**Authors:** Lindsay M. Reynolds, Timothy D. Howard, Carl D. Langefeld, Mara Z. Vitolins

**Affiliations:** 1 Department of Epidemiology and Prevention, Division of Public Health Sciences, Center for Precision Medicine, Wake Forest University School of Medicine, Winston-Salem, NC USA; 2 Department of Biochemistry, Center for Precision Medicine, Wake Forest University School of Medicine, Winston-Salem, NC USA; 3 Department of Biostatistics and Data Science, Division of Public Health Sciences, Center for Precision Medicine, and Center for Artificial Intelligence Research, Wake Forest University School of Medicine, Winston-Salem, NC USA

**Keywords:** Geroscience, Biological aging, Epigenetic aging, Dietary intervention, Mediterranean diet, Metabolic syndrome, Tree nuts, Extra virgin olive oil

## Abstract

Targeting biological processes of aging is a central goal of geroscience; however, limited data exist regarding the feasibility of incorporating biological aging biomarkers into dietary intervention trials. We conducted a pilot feasibility study among 34 adults aged 48–81 years with metabolic syndrome, a condition associated with elevated risk of age-related cardiometabolic disease and advanced biological aging. Participants consumed 1 oz of tree nuts and two tablespoons of extra virgin olive oil daily for 4 weeks. The primary objectives were to evaluate feasibility, adherence, and participant acceptability of epigenetic aging assessments. Exploratory outcomes included DunedinPACE, a measure of the pace of aging, and AgeAccelGrim, a measure of biological age relative to chronological age. At baseline, all participants exhibited a faster pace of biological aging than average as assessed by DunedinPACE, supporting metabolic syndrome as a promising target population for geroscience interventions. Adherence to the dietary intervention exceeded 95%, and most participants reported willingness to participate in a similar longer-term trial. Participants expressed a strong interest in learning their biological age and indicated that evidence of slowed aging would motivate sustained dietary change. No significant changes in epigenetic aging were observed over the 4-week intervention. These findings demonstrate the feasibility and acceptability of incorporating epigenetic aging biomarkers into dietary intervention research and suggest that biological aging measures may serve not only as surrogate outcomes but also as tools to support participant engagement. The results also support metabolic syndrome as a relevant population for dietary geroscience trials and provide practical guidance for designing longer-term studies evaluating whether dietary interventions can slow biological aging and promote healthy longevity. ClinicalTrials.gov Identifier: NCT04361617 (date of registration: 04-23-2020)

**Supplementary Information:**

The online version contains supplementary material available at https://doi.org/10.1007/s11357-026-02443-0.

## Introduction

Older age is the primary risk factor for most chronic diseases, including cardiovascular disease, type 2 diabetes, and neurodegenerative disorders [1, 2]. The geroscience hypothesis posits that interventions targeting fundamental biological processes of aging can simultaneously delay the onset or progression of multiple age-related conditions and extend healthspan [3–5]. Dietary interventions represent a promising, low‑risk strategy for targeting aging‑related disease pathways and promoting healthy aging. However, before large-scale dietary geroscience trials can be conducted, it is important to determine whether such interventions are feasible, whether participants will adhere to them, and whether biological aging measures can be successfully incorporated as study outcomes and participant engagement tools. Epigenetic aging biomarkers have emerged as promising indicators of biological aging and may help accelerate the evaluation of dietary interventions by providing surrogate measures of aging-related processes [6–9]. Therefore, the primary goal of this pilot study was to evaluate the feasibility of integrating epigenetic aging biomarkers into a simple dietary intervention, including evaluating participant adherence and perceptions of epigenetic aging information. A secondary goal was to generate preliminary data to inform the design of future dietary geroscience trials aimed at testing whether sustained dietary interventions can slow biological aging.

Multiple lines of research suggest that diet has potential to modify biological processes of aging and healthspan. Dietary patterns rich in unsaturated fats, plant‑based foods, and bioactive compounds—such as those characteristic of Mediterranean‑style diets—have been consistently associated with improved cardiometabolic health and reduced risk of age‑related diseases and mortality [10–14]. In a large, multicenter randomized controlled trial (PREDIMED), a Mediterranean‑style diet supplemented with extra virgin olive oil or mixed nuts, compared with a low‑fat control diet, reduced cardiovascular event rates over 5 years among 7447 individuals at high cardiovascular risk [10]. However, traditional clinical endpoints such as incidence of cardiovascular events require long follow‑up periods and large sample sizes, limiting their utility in a precision nutrition framework that accounts for individual factors known to influence response to diet, including life stage, health or disease status, and genetic factors [15, 16]. A growing body of work supports epigenetic aging biomarkers as geroscience-based outcomes capable of capturing short‑ to intermediate‑term changes in aging biology that may be feasible outcomes to inform novel precision nutrition-based approaches aiming to slow biological aging to increase healthspan.

DNA methylation–based epigenetic aging biomarkers have emerged as quantitative measures of biological aging which are predictive of healthspan, morbidity, and mortality [17, 18] and reflect the integrated effects of aging, genetics, environmental, and lifestyle factors [17–25]. These biomarkers offer several advantages for geroscience research, including applicability across the adult life course using minimally invasive biospecimens. Among these measures, DunedinPACE captures the rate or “pace” of biological aging [26], while GrimAge estimates biological age relative to chronological age and mortality risk [20]. Despite growing observational evidence linking diet quality to epigenetic aging [21–23, 27], relatively few intervention studies have incorporated these biomarkers into dietary trials [8, 28–30], and their integration into geroscience-guided study design remains underexplored.

Beyond their role as geroscience-based outcomes, epigenetic aging biomarkers may also function as personalized indicators of aging-related risk that motivate health behavior change. Whether individuals view biological age information as meaningful, acceptable, and actionable remains largely unknown, particularly in populations at elevated risk for age-related disease. Understanding participant perceptions is essential for translating aging biomarkers from research tools into components of patient-centered geroscience trials.

As geroscience interventions target biological age, the target population should also be defined based on biological age [31]. Individuals with metabolic syndrome may represent a biologically relevant population in which to test dietary geroscience trials. Metabolic syndrome is characterized by a cluster of cardiometabolic abnormalities that substantially increase risk for cardiovascular disease and type 2 diabetes, including abdominal obesity, high blood pressure, elevated fasting blood glucose, high triglycerides, and low high-density lipoprotein (HDL) cholesterol [32]. Metabolic syndrome is increasingly recognized as a state of advanced biological aging [33–39] that may be treated through lifestyle modifications including diet and exercise [40]. The PREDIMED dietary intervention involving extra virgin olive oil or mixed nut supplementation was more likely to improve cardiometabolic health and reverse metabolic syndrome compared to the low-fat control group in post hoc analysis [41]. However, lower levels of adherence to the PREDIMED intervention were found in participants with a higher number of cardiovascular risk factors, a larger waist circumference, and lower physical activity levels at baseline [42], highlighting the need for strategies that enhance engagement and sustain behavior change over time particularly in this population.

To inform trial design for future dietary geroscience trials, we conducted a 4-week pilot study of daily tree nut and extra virgin olive oil (EVOO) supplementation in adults with metabolic syndrome. The objectives of this study were to assess (1) the prevalence of advanced epigenetic aging in this high-risk population, (2) the adherence and feasibility of a simple dietary intervention involving EVOO and tree nut supplementation, (3) the acceptability of epigenetic aging assessments, and (4) the participant perceptions of biological age information as a potential motivator for sustained dietary behavior change. An exploratory objective was to quantify short‑term changes and variability in epigenetic aging biomarkers and metabolic syndrome components, as well as participant retention over the intervention period, to inform power calculations, sample size requirements, and feasibility assumptions for appropriately designing and powering future larger‑scale dietary geroscience trials aiming to slow the rate of biological aging and increase healthspan.

## Methods

### Study design and participants

This study was a prospective, longitudinal, two-arm feasibility pilot designed to inform the integration of biological aging biomarkers into dietary geroscience interventions. Men and women residing in and around Winston-Salem, North Carolina, were recruited.

Eligible participants were ≥ 35 years of age and met criteria for metabolic syndrome, defined as the presence of at least three of the following: waist circumference > 102 cm in men or > 88 cm in women; triglycerides > 150 mg/dL or treatment for hypertriglyceridemia; high-density lipoprotein cholesterol < 40 mg/dL in men or < 50 mg/dL in women or treatment for reduced high-density lipoprotein cholesterol; systolic blood pressure > 130 mm Hg or diastolic blood pressure > 85 mm Hg or antihypertensive treatment; fasting glucose > 100 mg/dL or hemoglobin A1c > 5.6% or use of oral hypoglycemic medications [32, 43].

Additional inclusion criteria included ability to comply with study procedures, ability to read and speak English, absence of allergies or hypersensitivities to tree nuts or olive oil, and ability to provide written informed consent. Exclusion criteria included plans to relocate within 12 weeks, body mass index (BMI) > 40 kg/m^2^, diagnosed or suspected dementia or cognitive impairment limiting protocol adherence, dietary practices likely to preclude adherence, homebound status, residence with another study participant, and insulin-dependent diabetes.

The study was approved by the Institutional Review Board of Wake Forest University Health Sciences (IRB00065273, date of approval 6/11/2020). Informed consent was obtained from all subjects involved in the study.

### Intervention and randomization

Participants meeting eligibility criteria were enrolled in a 4-week dietary intervention involving daily supplementation with tree nuts and extra virgin olive oil. At the intervention visit, participants were randomized in a 1:1 allocation to receive education about epigenetic aging or to receive no such education. Participants randomized to the epigenetic aging education arm received written educational materials and a brief standardized explanation of epigenetic aging (Supplemental Materials). All participants provided blood samples for epigenetic aging assessment.

### Intervention foods

All participants received a 4-week supply of extra virgin olive oil (EVOO) and tree nuts, consisting of individual 1-oz daily servings of unsalted English walnuts, almonds, and pistachios (approximately 10 days’ supply of each nut type). Participants were instructed to consume 1 oz (29 g) of tree nuts and two tablespoons of extra virgin olive oil daily by incorporating these foods into their habitual diets. Educational materials and recipes were provided to support substitution for other dietary fats or snack foods. Daily dietary adherence diaries were used to track intake of the study foods.

Intervention foods were based in part on the PREDIMED trial intervention in which participants were instructed to consume either 30 g of mixed nuts per day (15 g walnuts, 7.5 g hazelnuts, and 7.5 g almonds) or 4 tablespoon of EVOO per day [10]. Because both of the EVOO and mixed nut intervention arms of PREDIMED were separately associated with reversion of metabolic syndrome compared to the low-fat diet control arm [41], we choose to combine both EVOO and nuts into one intervention arm. To avoid increasing caloric intake and promoting weight gain, we reduced the EVOO component to 2 tablespoons per day, which was aligned with the FDA statement that “*Limited and not conclusive scientific evidence suggests that eating about 2 tablespoons (23 g) of olive oil daily may reduce the risk of coronary heart disease due to the monounsaturated fat in olive oil” *[44]*.*

### Study procedures and measures

In-person study visits were conducted at baseline and at the end of the 4-week intervention. At baseline, participants completed questionnaires assessing demographic characteristics, health history, and adherence to a Mediterranean-style dietary pattern [45], including baseline nut and olive oil consumption. After a minimum 5-min seated rest, blood pressure was measured. Body weight (kg), height (cm), and waist circumference (cm) were measured using standardized protocols.

Fasting blood samples were collected for measurement of triglycerides, high-density lipoprotein cholesterol, and fasting glucose through a certified clinical laboratory. Additional fasting blood was collected in EDTA tubes for DNA methylation analyses conducted at Wake Forest University School of Medicine.

Telephone follow-up visits were conducted at weeks one and two to assess safety, discuss potential side effects, review adherence diaries, and provide dietary counseling as needed. At the final week-four visit, all baseline measurements and questionnaires were repeated, and fasting blood samples were collected.

To evaluate feasibility and participant experience, all participants completed an Intervention Experience Assessment at the final visit. Participants in the epigenetic aging education arm additionally completed an exit questionnaire assessing perceptions of epigenetic aging information and its potential motivational impact on dietary behavior.

### Epigenetic aging assessment

DNA was extracted from fasting whole blood samples and bisulfite converted using the EZ DNA Methylation Gold kit (Zymo Research). Genome-wide DNA methylation was measured using the Illumina Infinium MethylationEPIC BeadChip, interrogating over 850,000 CpG sites, and scanned using the Illumina iScan system.

Quality control procedures included verification that cumulative probe signal exceeded negative control thresholds and retention of probes with detection *p* values ≤ 0.05 in at least 90% of samples. Data processing and normalization were performed using the Bioconductor package *ChAMP* [46], including BMIQ normalization for Infinium I and II probe types. Batch effects related to BeadChip were adjusted using ComBat [47]. After quality control, 720,027 CpG sites were retained for analysis.

Epigenetic aging measures were derived from normalized DNA methylation beta values using DunedinPACE [26] and GrimAge [20] algorithms. DunedinPACE estimates the rate of biological aging, with values > 1 indicating faster-than-average aging. GrimAge was regressed on chronological age to generate estimates of the difference in epigenetic age relative to chronological age (AgeAccelGrim), with values > 0 indicating advanced biological age relative to chronological age.

### Outcomes

At baseline, the prevalence of advanced epigenetic aging was assessed, defined as DunedinPACE greater than 1 and AgeAccelGrim greater than 0. Adherence to daily consumption of tree nuts and extra virgin olive oil was based on participant daily adherence diaries and was quantified as the percent of days tree nuts, extra virgin olive oil, or both were reported to be consumed. Retention was defined as percent of eligible participants that completed the 4-week intervention. Other feasibility outcomes included participant-reported difficulty with adherence to the tree nuts and EVOO which was assessed using a Likert scale ranging from “not difficult at all” to “very difficult” and participant willingness/ability to participate in a longer-term study like this one lasting 3–4 years (yes or no) as detailed in the Supplemental Materials. Participant interest in knowing their epigenetic age was assessed using a Likert scale ranging from “not at all” to “very much.” Perceived motivational impact of biological aging information on adherence to the dietary intervention was assessed using a Likert scale ranging from “very unlikely” to “very likely.” Exploratory outcomes examined included changes in epigenetic aging biomarkers and metabolic syndrome components from baseline to 4 weeks.

### Statistical analysis

Descriptive statistics were used to summarize participant characteristics, adherence, retention, and feasibility outcomes. Advanced epigenetic aging was evaluated using one-sided Wilcoxon tests comparing DunedinPACE values against 1.0 and GrimAge acceleration values against 0. Paired *t* tests were used to compare baseline and post-intervention values for epigenetic aging biomarkers and metabolic syndrome components. The study was not powered to detect intervention effects. All analyses were performed in R version 4.5.2.

## Results

### Participant enrollment and baseline characteristics

Participants were enrolled between July 2021 and February 2022. Of 54 individuals who attended in-person screening, 53 provided informed consent, and 34 (63%) met eligibility criteria for metabolic syndrome and were enrolled. Among excluded individuals, 17 did not meet metabolic syndrome criteria and two had a body mass index greater than 40 kg/m^2^. One participant withdrew prior to study completion, resulting in 33 participants completing the 4-week intervention (97% retention).

Participants ranged in age from 48 to 81 years [mean ± standard deviation (SD): 68 ± 9 years] and were randomized to either an epigenetic aging education arm or an active comparator arm (Table 1). The majority of participants were female (59%), White (56%), and non-Hispanic (100%). Baseline adherence to a Mediterranean-style dietary pattern was low to moderate (mean MedDiet Screener score ± SD: 6.7 ± 1.9 out of a maximum of 14 points). Olive oil was reported as the primary culinary fat by 45% of participants, with mean ± SD baseline consumption of 0.9 ± 3.5 tablespoons per day. Mean ± SD baseline nut consumption, including peanuts, was 6.2 ± 4.5 servings per week.

**Table 1:** Population characteristics

Characteristic	Overall *N* = 34	Epigenetic age knowledge arm *N* = 17	Active comparator arm *N* = 17
Age mean (SD)	68 (9)	65 (11)	70 (8)
Gender			
Female, *N*	20	12	8
Male, *N*	14	5	9
Race			
Black or African American, *N*	13	6	7
White, *N*	19	11	8
More than one race, *N*	1	0	1
Unknown/not reported, *N*	1	0	1
Ethnicity: non-Hispanic, *N*	34	17	17
Education			
High school, *N*	3	2	1
> High school, < Bachelors degree, *N*	13	6	7
Bachelors degree, *N*	11	7	4
Post-graduate degree, *N*	6	1	5
MedDiet Screener score			
Weak adherence (0–5 points)	7	3	4
Moderate adherence (6–9 points)	24	13	11
Good adherence (10–14 points)	2	1	1
Epigenetic aging measures			
DunedinPACE, mean (SD)	1.179 (0.089)	1.146 (0.075)	1.208 (0.092)
AgeAccelGrim, mean (SD)	0.06 (2.24)	−0.51 (1.82)	0.56 (2.50)

MedDiet Screener score reflects adherence to a Mediterranean-like diet [45]

### Baseline epigenetic aging profile

**Fig. 1:**
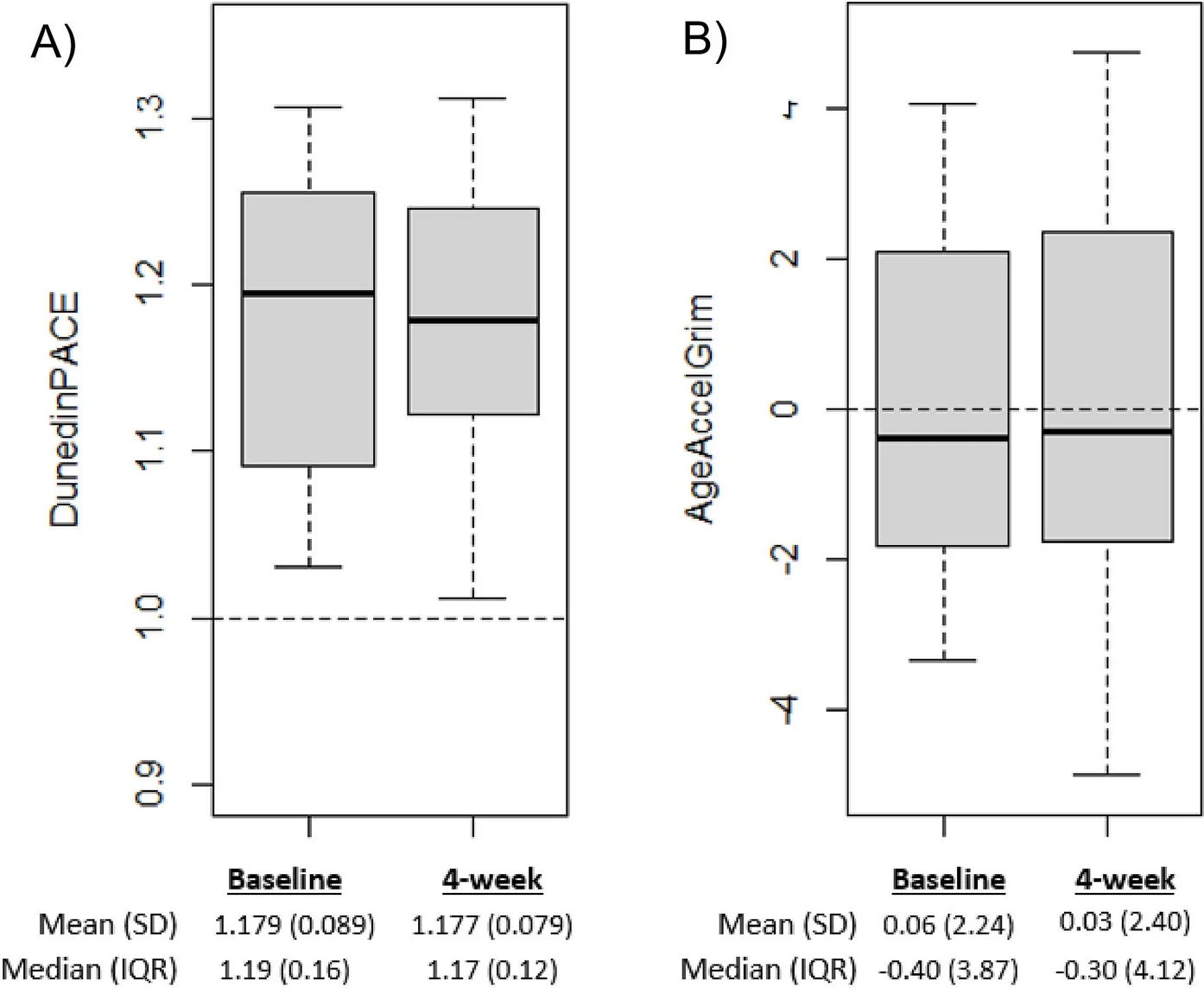
Epigenetic aging biomarkers measured in adults with metabolic syndrome before and after a 4-week dietary intervention involving daily consumption of 1 oz of tree nuts and two tablespoons of extra virgin olive oil. Boxplots depict the median, interquartile range, and range (*y*-axis) of two epigenetic aging biomarkers: DunedinPACE and AgeAccelGrim. **A** DunedinPACE represents a measure of the current pace of biological aging, with values > 1 indicating faster biological aging than average (e.g., a value of 1.10 indicates a pace of aging 10% faster than the average). **B** AgeAccelGrim represents the difference in biological age relative to chronological age, with values > 0 indicating advanced biological age relative to chronological age (e.g., a value of 1 indicates a biological age that is 1 year older than chronological age)

At baseline, participants exhibited advanced biological aging as measured by DunedinPACE, an estimate of the pace of aging. All participants (100%) demonstrated a pace of aging faster than 1 year of biological aging per year of chronological aging (DunedinPACE mean ± SD: 1.18 ± 0.09; Wilcoxon test vs 1.0, *p* = 3.73 × 10⁻⁹; Fig. 1A). In contrast, advanced biological age relative to chronological age as measured by AgeAccelGrim was not consistently observed, with 11 of 29 participants showing positive AgeAccelGrim values (Wilcoxon test vs 0, *p* = 0.48). These findings indicate substantial heterogeneity across epigenetic aging biomarkers and suggest that DunedinPACE may be particularly sensitive to biological aging in adults with metabolic syndrome.

### Intervention adherence and feasibility

Adherence to daily supplementation with tree nuts and extra virgin olive oil was high across the intervention period. The majority (81%) of participants reported that consuming 1 oz of tree nuts daily was “not difficult at all” with mean adherence of 98.6%. Consumption of two tablespoons of olive oil daily was reported as between “not difficult at all” and “moderately difficult,” with mean adherence of 96.4%. Most participants (84%) reported they would be able to continue daily consumption of tree nuts and olive oil in a similar study lasting 3 to 4 years. Detailed results from the post-intervention feasibility and experience survey are provided in the Supplemental Materials.

### Participant perceptions of epigenetic aging

Participants randomized to receive epigenetic aging education were asked to complete an exit questionnaire assessing perceptions of epigenetic aging information and its potential motivational impact on dietary behavior. Among the participants that completed the exit questionnaire, 77% reported that they “very much” wanted to know their epigenetic age. Additionally, 82% reported that if informed their biological age was more advanced than their chronological age, they would feel “more motivated” or “very motivated” to adhere to the dietary intervention. These findings suggest that biological aging information was perceived as meaningful and potentially actionable by participants at elevated cardiometabolic risk.

### Exploratory changes in epigenetic aging and metabolic measures

**Fig. 2:**
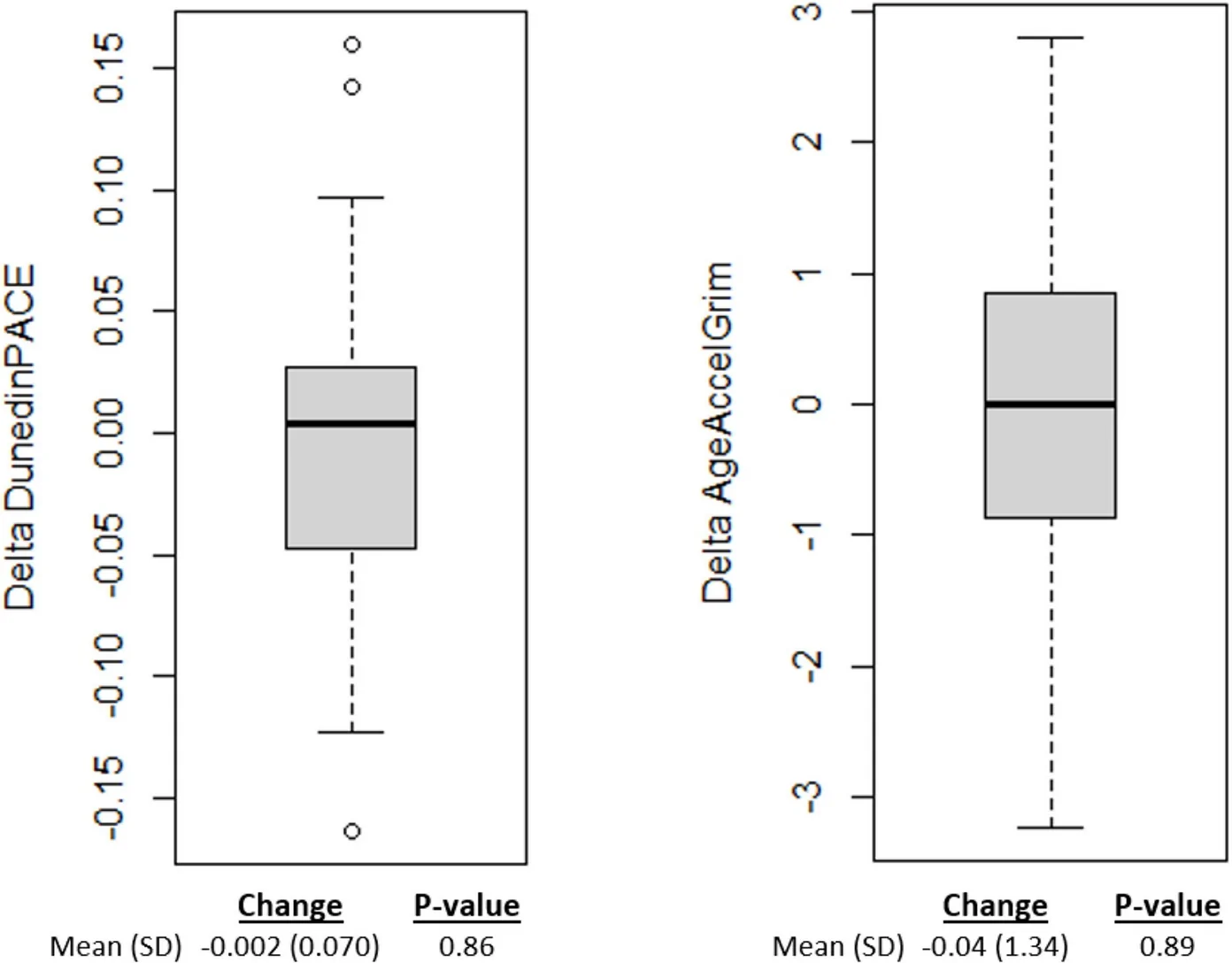
Change in epigenetic aging biomarkers among adults with metabolic syndrome following a 4-week dietary intervention. Boxplots depict the median, interquartile range, and range (*y*-axis) of the change in DunedinPACE and AgeAccelGrim over the 4-week intervention. **A** Delta DunedinPACE represents the change in the pace of biological aging over 4 weeks, with values < 0 indicating a slower pace of aging following the intervention (e.g., a value of − 0.05 indicates a pace of aging that is 5% slower at follow-up than at baseline). **B** Delta AgeAccelGrim represents the change in biological age relative to chronological age over 4 weeks, with values < 0 indicating a younger biological age relative to chronological age following the intervention (e.g., a value of − 1 indicates a biological age that is 1 year younger relative to chronological age at follow-up than at baseline). Data are shown for the 29 participants with DNA methylation measures available at both time points. Statistical significance of within-participant change was assessed using paired *t* tests

**Fig. 3:**
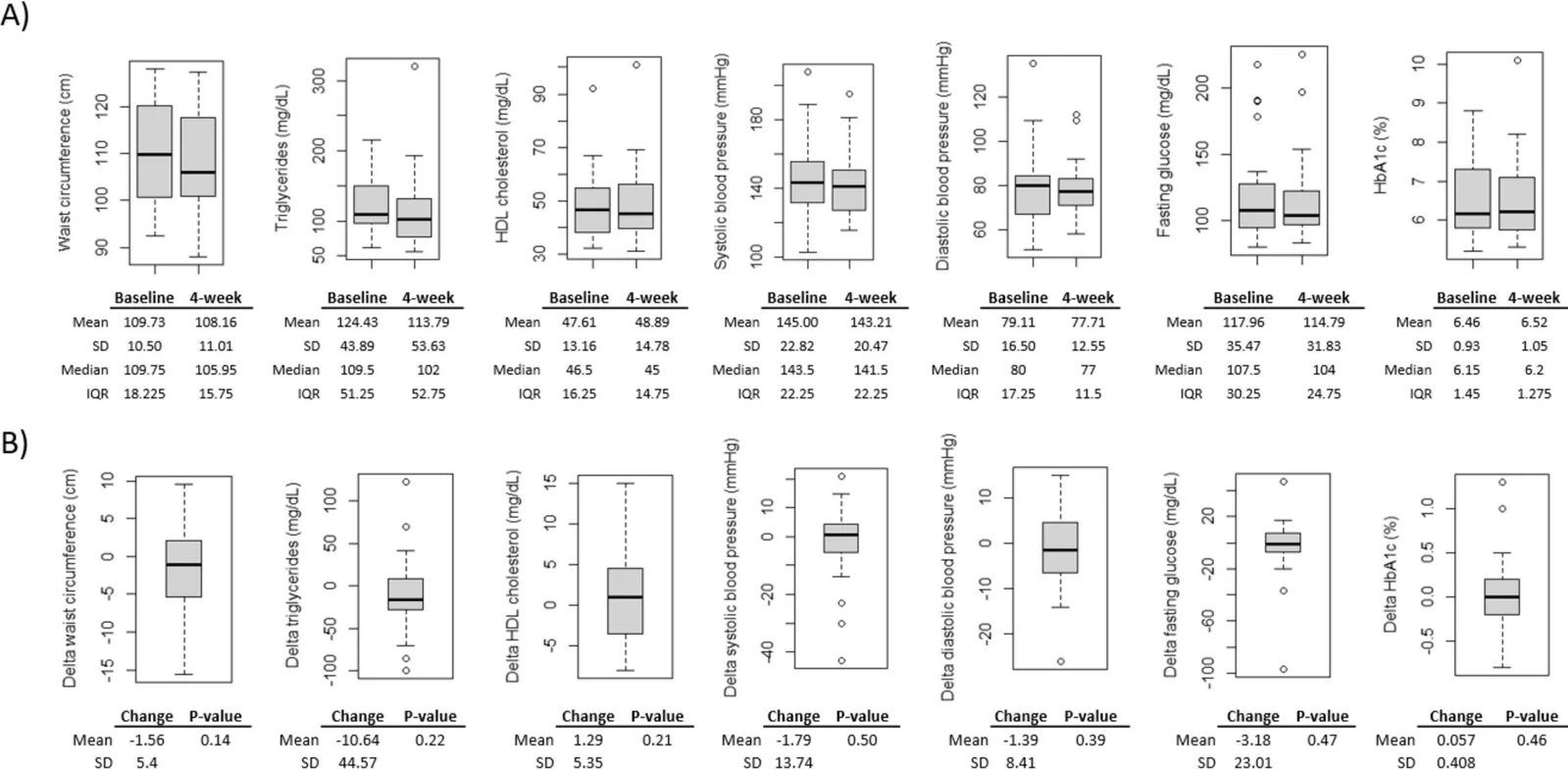
Metabolic syndrome component measures before and after a 4-week dietary intervention. **A** Boxplots depict the median, interquartile range, and range (*y*-axis) of metabolic syndrome components measured at baseline and at the end of the intervention among 33 participants with metabolic syndrome who consumed 1 oz of tree nuts and two tablespoons of extra virgin olive oil daily. **B** Boxplots depict the median, interquartile range, and range (*y*-axis) of within-participant change in metabolic syndrome component measures from baseline to the end of the 4-week intervention. Statistical significance of change was assessed using paired *t* tests; *p* values are shown for each component

On average, both DunedinPACE and AgeAccelGrim values decreased modestly from baseline to the end of the 4-week intervention; however, these changes were not statistically significant (Fig. 2). Similarly, metabolic syndrome component measures, including waist circumference, lipid levels, blood pressure, and fasting glucose, showed trends toward improvement with the exception of hemoglobin A1c, though none of these changes reached statistical significance over the short intervention period (Fig. 3).

## Discussion

This feasibility study represents an initial step toward integrating a simple dietary intervention with biological aging biomarkers in geroscience-guided clinical research. Specifically, the study contributes evidence supporting adults with metabolic syndrome as a promising target population for dietary geroscience trials and demonstrates high participant adherence to a simple dietary intervention and strong interest in epigenetic aging assessments. By combining a tree nut and extra virgin olive oil supplementation intervention with epigenetic aging assessments in adults with metabolic syndrome, this study was designed to evaluate the feasibility and acceptability of incorporating biological aging measures into dietary interventions and to provide practical information to inform the design of future dietary interventions aimed at slowing biological aging.

A central finding of this pilot study is the high prevalence of advanced biological aging among adults with metabolic syndrome, particularly when assessed using DunedinPACE. Together with previous studies reporting associations between metabolic syndrome and advanced epigenetic aging [39, 48, 49], these findings further support the concept that individuals with metabolic syndrome may be an attractive target for geroscience interventions. Because a faster rate of biological aging may provide greater opportunity for measurable improvement, interventions conducted in this population may be more sensitive for detecting effects on biological aging than studies conducted in healthier populations.

The findings also highlight important differences among epigenetic aging biomarkers and have implications for biomarker selection in future dietary geroscience trials. All participants exhibited a pace of aging faster than average based on DunedinPACE, but older biological ages relative to chronological ages as assessed by GrimAge were not consistently observed. These differences likely reflect the distinct biological constructs captured by the biomarkers. DunedinPACE was developed to measure the current rate of physiological decline and was trained using longitudinal changes in multiple cardiometabolic parameters, including waist-to-hip ratio, blood pressure, HbA1c, triglycerides, HDL cholesterol, and total cholesterol. In contrast, GrimAge primarily reflects mortality risk through DNAm-based surrogate measures of plasma proteins and smoking history. DunedinPACE also differs conceptually from first- and second-generation epigenetic clocks because it estimates the pace of aging rather than cumulative aging burden. Observational evidence, including analyses from the Women’s Health Initiative, suggests that diet quality may be more strongly associated with DunedinPACE than with other epigenetic aging measures [23]. Collectively, these findings support DunedinPACE as a promising candidate biomarker for dietary geroscience research, although larger longitudinal studies are needed to evaluate its responsiveness to intervention and its role as a mediator of diet-healthspan relationships.

An additional contribution of this study is the demonstration that epigenetic aging assessments were highly acceptable to participants and perceived as meaningful and motivating. Most individuals expressed strong interest in learning their biological age and reported that evidence of slowed aging would motivate long-term dietary change. These findings are particularly relevant because maintaining adherence is a major challenge in long-term dietary interventions, especially among individuals with multiple cardiometabolic risk factors [42]. The present findings suggest that biological aging assessments may serve not only as surrogate outcomes but also as engagement tools that enhance participant motivation and support sustained behavior change. However, the effects of biological age feedback on long-term adherence and lifestyle behaviors require further investigation.

Importantly, this feasibility study demonstrated high adherence to a simple dietary intervention involving supplementation with 1 oz of tree nuts and two tablespoons of extra virgin olive oil per day among adults with metabolic syndrome. Supporting selection of tree nuts and extra virgin olive oil as components of a geroscience-oriented dietary intervention, consumption of nuts and extra virgin olive oil have each been associated with anti-inflammatory and cardiometabolic benefits [50–53], including reversion of metabolic syndrome when compared with a low-fat diet control, intervention [41]. Further, both foods contain nutrients and bioactive compounds that may influence biological pathways involved in aging. Tree nuts are highly nutritious foods which provide unsaturated fatty acids (oleic, linoleic, and alpha-linolenic acids) and bioactive compounds including vitamin E, carotenoids, phytosterols, and polyphenols [51]. Dietary olive oil has been linked with benefits on virtually all of the hallmarks of aging, which may be attributable to its monounsaturated fatty acid (oleic acid) content and minor bioactive components including phenolic compounds [52]. The high adherence observed in this study supports the feasibility and acceptability of this combined intervention for future longer-term geroscience trials designed to evaluate its effects on biological aging.

This feasibility study generated essential data on participant adherence, biomarker variability, and participant engagement that will directly inform intervention design, sample size calculations, and outcome selection for future longer-term dietary geroscience trials. As expected, this 4-week intervention did not result in statistically significant changes in epigenetic aging. However, the primary objective of this study was not to evaluate intervention efficacy but rather to determine whether epigenetic aging biomarkers could be successfully integrated into a dietary intervention and whether such assessments were acceptable and meaningful to participants at elevated risk for age-related cardiometabolic disease.

Several factors should be considered when interpreting the absence of detectable changes in epigenetic aging. DNA methylation–based biomarkers are subject to both technical and biological variability, and within-person variation may be substantial even over short time periods [54]. Consequently, the ability to detect modest intervention-related changes depends heavily on the signal-to-noise ratio. In addition, the intervention consisted of supplementation layered onto participants’ habitual diets rather than a comprehensive dietary overhaul. While this intervention was selected based on evidence supporting favorable effects on cardiometabolic health and biological pathways linked to aging, the magnitude of change in the rate of biological aging expected over the 4-week intervention period is modest.

Importantly, studies reporting significant reductions in epigenetic aging have generally involved substantially larger sample sizes and intervention durations exceeding 1 year [7, 8, 29]. Thus, the absence of observable changes in the present study should not be interpreted as evidence that dietary interventions are incapable of influencing biological aging. Rather, it likely reflects the combined effects of the short intervention duration, modest expected effect size, limited sample size, and inherent variability in epigenetic aging measures.

Taken together, these findings help build a foundation for future dietary geroscience trials. The findings support adults with metabolic syndrome as a target population characterized by faster biological aging than average, demonstrate strong participant adherence and acceptability of epigenetic aging assessments, and highlight potential advantages of DunedinPACE as an intervention biomarker. Future studies should evaluate whether longer-duration dietary interventions and larger sample sizes can slow biological aging in populations characterized by advanced biological aging. Such studies may be particularly informative because individuals with faster biological aging may have greater potential for improvement and may provide a more sensitive context for detecting intervention-related changes in biological age.

In addition to the small sample size and short intervention duration, several other limitations should be considered when interpreting these findings. Adherence was assessed using self-reported adherence diaries. Future studies would be strengthened by incorporating objective dietary intake biomarkers, such as circulating fatty acid profiles and olive oil–derived polyphenol metabolites, to verify adherence to tree nut and extra virgin olive oil consumption. Also, we assessed adherence to a Mediterranean-style dietary pattern [45], including baseline nut and olive oil consumption, but we did not assess changes in diet quality that may occur in parallel with increased tree nut and olive oil consumption. Interpreting results from future geroscience dietary trials would be enhanced by including repeated comprehensive dietary assessments such as multiple Automated Self-Administered 24-h recalls (ASA24s) [55] to capture potential changes in diet quality. Also, the absence of a placebo or control diet limits conclusions regarding efficacy. Additionally, participant perceptions of epigenetic aging were assessed qualitatively and warrant more rigorous evaluation in larger studies. Despite these limitations, the study demonstrates strong feasibility, high adherence, and substantial participant interest in biological aging measures.

In summary, this study demonstrates the feasibility and acceptability of integrating epigenetic aging biomarkers into a simple dietary intervention among adults with metabolic syndrome. The findings support metabolic syndrome as a promising target population for dietary geroscience interventions and identify DunedinPACE as a potentially informative biomarker for future studies. Participant recruitment and retention were successful, adherence to the intervention was high, and participants expressed strong interest in biological aging measures, highlighting their potential to enhance engagement in future trials. Collectively, these results advance the integration of biological aging measures into nutrition research and provide practical guidance for designing future geroscience-informed dietary interventions, including target population selection, biomarker choice, and participant engagement strategies.

## Supplementary Information

Below is the link to the electronic supplementary material.

ESM 1 Supplementary Material 1 (PDF 474 KB)

## Data Availability

De-identified data are available upon reasonable request.
