# Supplementary material for: Epigenetic aging biomarkers in dietary geroscience: feasibility, participant perceptions, and trial design considerations
Source: GeroScience. 2026 Jul 29;48(5):6251–62. doi: 10.1007/s11357-026-02443-0 (PMC13601483; doi:10.1007/s11357-026-02443-0)
Supplement: Supplementary file 1 [file 11357_2026_2443_MOESM1_ESM.pdf]

Supplementary Materials

Epigenetic Aging Biomarkers in Dietary Geroscience: Feasibility, Participant Perceptions, and Trial Design Considerations

Table of Contents

Epigenetic Aging Educational Information

Modified 14-Item Questionnaire of Mediterranean diet adherence

Participant Experiences Interview

Epigenetic Aging Exit Questionnaire

Epigenetic Aging Educational Information

Nuts & Oils - Epigenetic Aging

Eating more extra virgin olive oil and tree nuts (walnuts, almonds, and pistachios) may improve your metabolic health. We want to learn more about the positive effects that increasing tree nuts (walnuts, almonds, and pistachios) and extra virgin olive oil consumption can have on your body. To do this, we are examining markers in your blood and are testing for potential benefits of nuts and oils on your body as it ages. We are interested in how this simple adjustment to your diet might improve how your body ages by measuring the difference between how old you are (the number of years since you were born) and how old the cells in your body behave (biological age). The marker of biological aging we are measuring in blood is known as "epigenetic age".

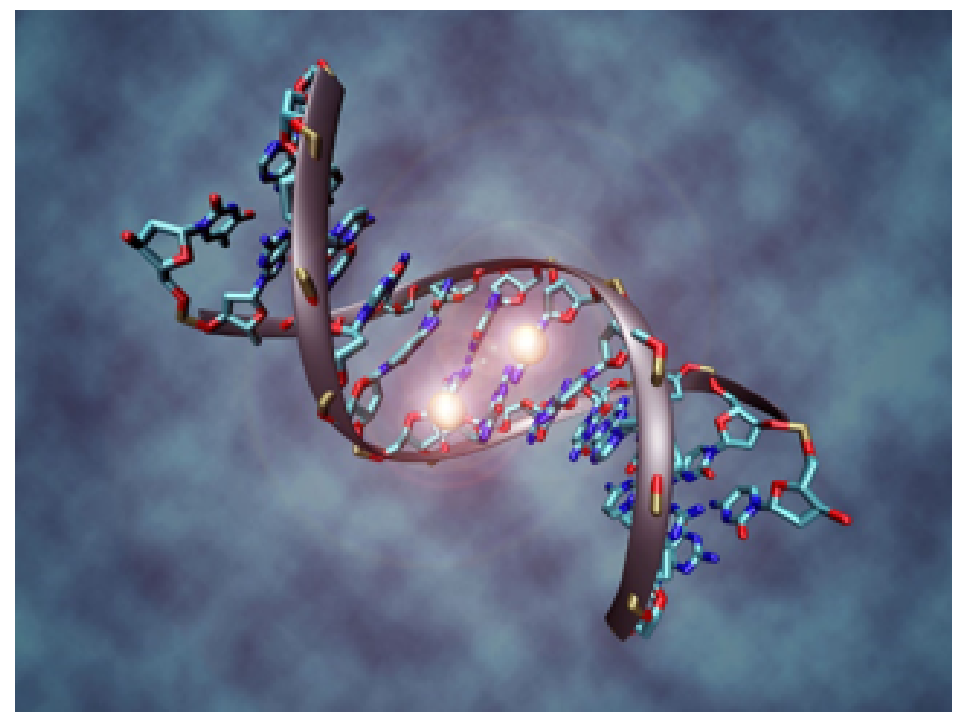

Epigenetic age is a measure of predicted biological age, based on DNA methylation. DNA methylation is a way that your body controls which genes are expressed in a specific tissue. DNA methylation is reversible and can play a role in turning genes on and off.

Diet, obesity, diabetes and other conditions can be related to increased epigenetic age relative to chronological age, known as **epigenetic age acceleration**. Epigenetic age acceleration can be used to predict lifespan, as well as the risk of cardiovascular disease and death. However, more research is needed to better understand the way that lifestyle changes can impact epigenetic aging.

We believe that eating more nuts and olive oil may improve your metabolic health and slow biological aging. To test if we are right, we will compare your epigenetic age measured before and after 4 weeks of eating nuts (1 oz daily) and olive oil (2 tablespoons daily). Measuring differences in epigenetic age before and after 4 weeks of eating more nuts and oil will help us to better understand the potential for nuts and oil to slow biological aging. It may also help us to identify who may benefit most from adding nuts and oils to their diets.

Please let us know if you have any questions about your epigenetic age.

MEDITERRANEAN DIET SCREENER – A modified 14-Item Questionnaire of Mediterranean diet adherence to assess baseline diet including consumption of olive oil and nuts[27,28].

1. **Do you use olive oil as your main cooking fat?** (Criteria for 1 point: Yes)

   1☐ Yes

   2☐ No

2. **How much olive oil do you consume in a given day (including oil used for frying, salads, restaurant meals, etc.)?** (Criteria for 1 point: ≥4)

   ☐☐ tablespoons

3. **How many vegetable servings do you consume per day? (1 serving is ½ a cup of cooked vegetables or 1 cup of raw vegetables)** (Criteria for 1 point: ≥2)

   ☐☐ servings

4. **How many fruit servings, including natural fruit juices, do you consume per day? (1 serving is a medium fresh fruit; ½ cup of chopped, cooked, or canned fruit; ½ cup of juice)** (Criteria for 1 point: (Criteria for 1 point: ≥3)

   ☐☐ servings

5. **How many servings of red meat, hamburger, or meat products (ham, sausage, etc.) do you consume per day? (1 serving is 2-3 ounces)** (Criteria for 1 point: <1)

   ☐☐ servings

6. **How many servings of butter, margarine, or cream do you eat per day? (1 serving is 1 Tbsp.)** (Criteria for 1 point: <1)

   ☐☐ servings

7. **How many sweet or carbonated beverages do you drink per day?** (Criteria for 1 point: <1)

   ☐☐ beverages

8. **How much wine do you drink per week?** (Criteria for 1 point: ≥7)

   ☐☐ glasses

9. **How many servings of legumes do you consume per week? (1 serving is ½ cup of beans or lentils)** (Criteria for 1 point: ≥3)

☐☐ servings

10. **How many serving of fish or shellfish do you consume per week? (1 serving is 2-3 ounces)** (Criteria for 1 point: ≥3)

☐☐ servings

11. **How many times per week do you consume commercial sweets or pastries (not homemade), such as cakes, cookies, pies, or pudding?** (Criteria for 1 point: <3)

☐☐ times

12. **How many servings of nuts (including peanuts) do you consume per week? (1 serving is ¼ cup of nuts or 2 tablespoons of nut butter)** (Criteria for 1 point: ≥3)

☐☐ servings

13. **Do you preferentially consume chicken, turkey, or rabbit meat instead of veal, pork, hamburger, or sausage?** (Criteria for 1 point: Yes)

    1☐ Yes

    2☐ No

14. **How many times per week do you consume vegetables, pasta, rice, or other dishes seasoned with sauce made with tomato and onion, leek, or garlic and simmered with olive oil?** (Criteria for 1 point: ≥2)

☐☐ times

Participant Experiences Interview – Results from the participant exit survey conducted after the 4-week tree nut and EVOO intervention:

a) On a scale of 1 to 10, how difficult did you find it to eat the tree nuts and olive oil every day?
Counts/frequency:
1 Not Difficult at all (5, 45.5%)
2 (1, 9.1%)
3 (1, 9.1%)
4 (2, 18.2%)
5 Moderately Difficult (0, 0.0%)
6 (1, 9.1%)
7 (0, 0.0%)
8 (1, 9.1%)
9 (0, 0.0%)
10 Very Difficult (0, 0.0%)

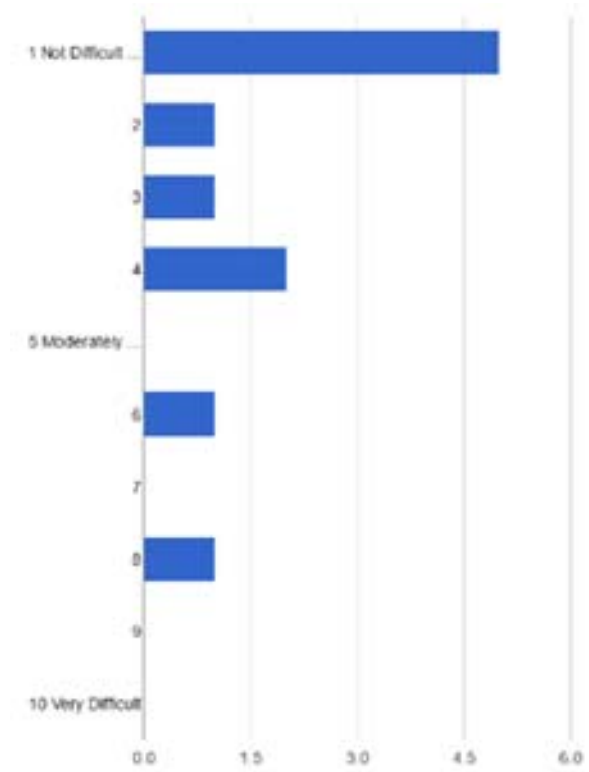

b) On a scale of 1 to 10, how difficult did you find it to eat the tree nuts every day?
Counts/frequency:
1 Not Difficult at all (17, 81.0%)
2 (3, 14.3%)
3 (0, 0.0%)
4 (0, 0.0%)
5 Moderately Difficult (0, 0.0%)
6 (1, 4.8%)
7 (0, 0.0%)
8 (0, 0.0%)
9 (0, 0.0%)
10 Very Difficult (0, 0.0%)

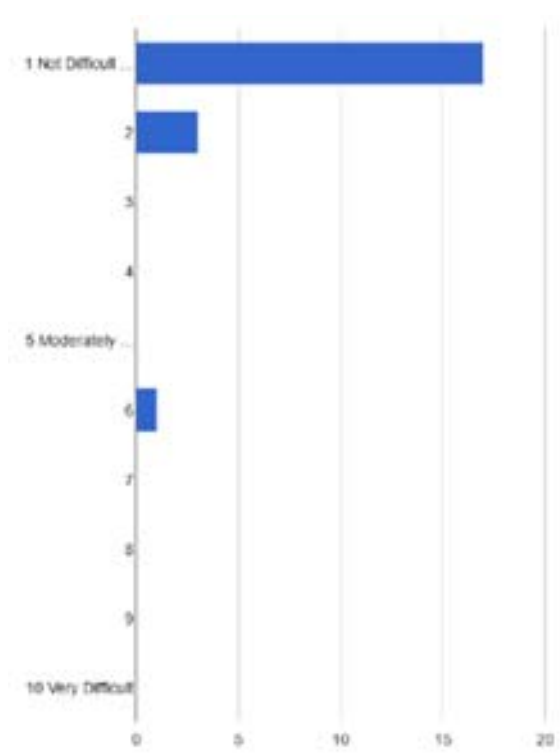

c) On a scale of 1 to 10, how difficult did you find it to consume the olive oil every day?
Counts/frequency:
1 Not Difficult at all (7, 33.3%)
2 (2, 9.5%)
3 (4, 19.0%)
4 (4, 19.0%)
5 Moderately Difficult (1, 4.8%)
6 (1, 4.8%)
7 (0, 0.0%)
8 (2, 9.5%)
9 (0, 0.0%)
10 Very Difficult (0, 0.0%)

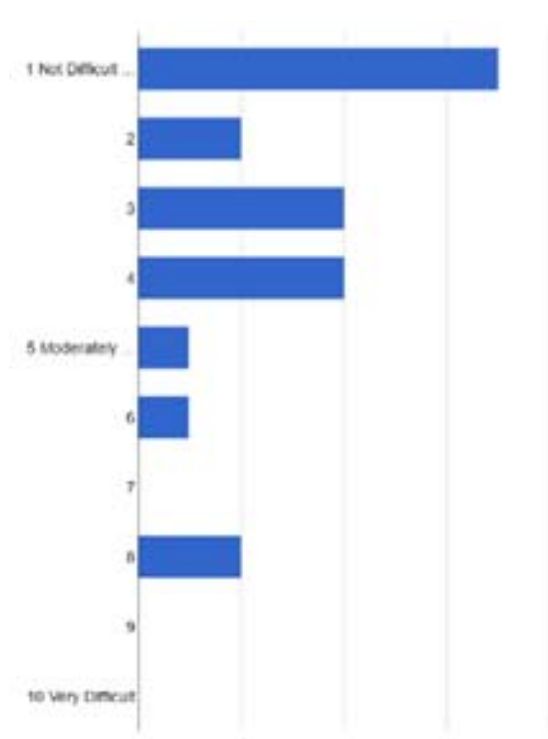

d) On a scale of 1 to 10, how did eating the tree nuts and olive oil daily make you feel?

Counts/frequency:

1 Not Good At All (0, 0.0%)
2 (0, 0.0%)
3 (1, 8.3%)
4 (1, 8.3%)
5 No Difference (4, 33.3%)
6 (2, 16.7%)
7 (1, 8.3%)
8 (1, 8.3%)
9 (1, 8.3%)
10 Very Good (1, 8.3%)

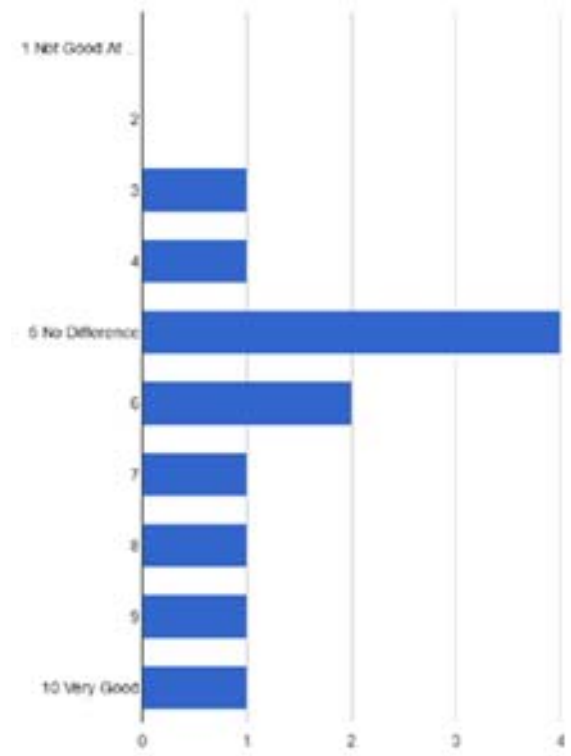

e) On a scale of 1 to 10, how did eating the tree nuts daily make you feel?

Counts/frequency:

1 Not Good At All (0, 0.0%)
2 (0, 0.0%)
3 (0, 0.0%)
4 (0, 0.0%)
5 No Difference (10, 47.6%)
6 (3, 14.3%)
7 (0, 0.0%)
8 (0, 0.0%)
9 (1, 4.8%)
10 Very Good (7, 33.3%)

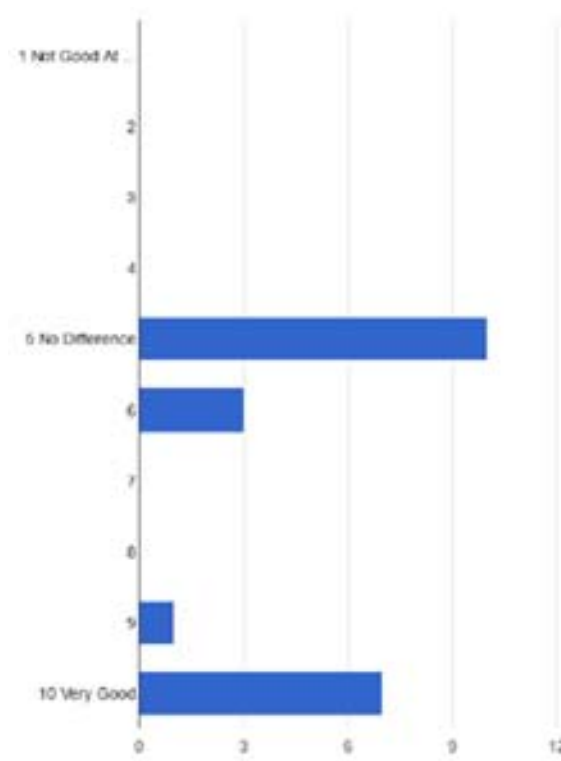

f) On a scale of 1 to 10, how did consuming the olive oil daily make you feel?

Counts/frequency:

1 Not Good At All (0, 0.0%)
2 (2, 9.5%)
3 (0, 0.0%)
4 (2, 9.5%)
5 No Difference (10, 47.6%)
6 (4, 19.0%)
7 (1, 4.8%)
8 (0, 0.0%)
9 (0, 0.0%)
10 Very Good (2, 9.5%)

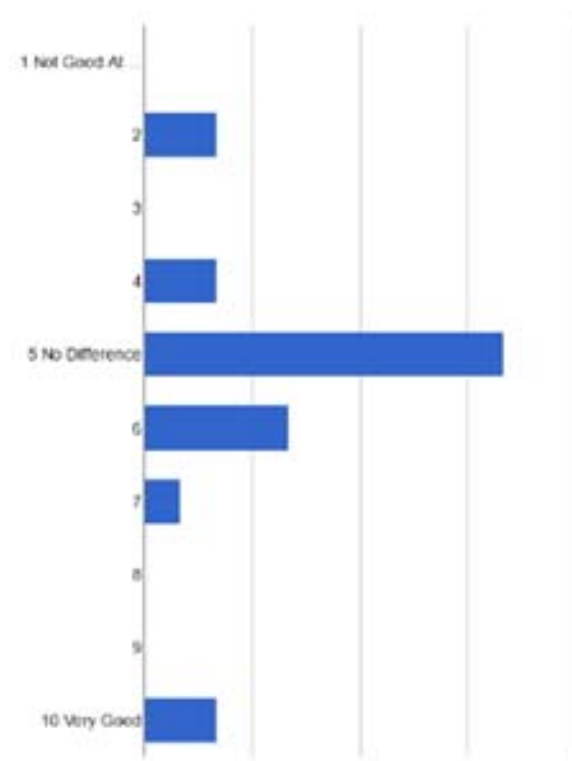

g) Will you continue to eat tree nuts and olive oil daily after completing the research study?

Counts/frequency:

Very unlikely (0, 0.0%)
Unlikely (0, 0.0%)
Neutral (2, 16.7%)
Somewhat likely (3, 25.0%)
Very likely (7, 58.3%)

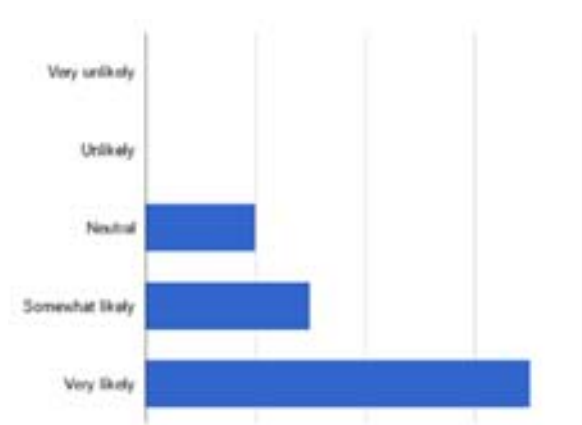

h) Will you continue to eat tree nuts daily after completing the research study?

Counts/frequency:

Very unlikely (1, 4.8%)
Unlikely (1, 4.8%)
Neutral (1, 4.8%)
Somewhat likely (7, 33.3%)
Very likely (11, 52.4%)

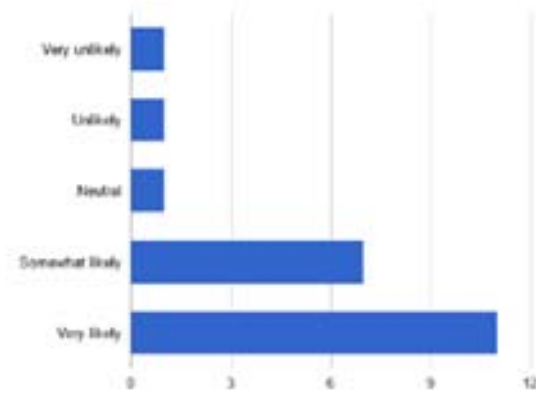

i) Will you continue to eat olive oil daily after completing the research study?

Counts/frequency:

Very unlikely (1, 4.8%)
Unlikely (2, 9.5%)
Neutral (2, 9.5%)
Somewhat likely (10, 47.6%)
Very likely (6, 28.6%)

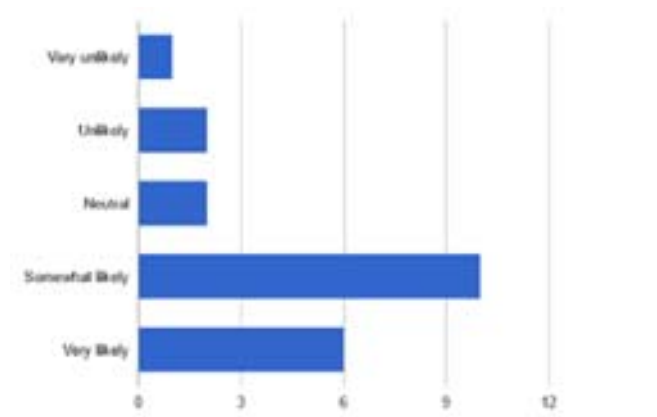

j) Do you think that you would be able to participate in a study like this one that lasted 3-4 years? In other words, would you be able to continue eating the tree nuts and olive oil for 3-4 years?

Counts/frequency:

Yes (27, 84.4%)
No (5, 15.6%)

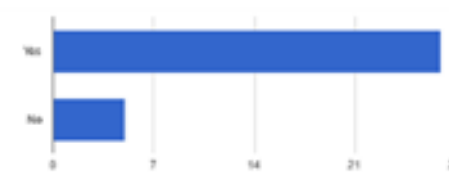

Epigenetic Aging Exit Questionnaire Results

1. **Using the scale below, indicate how much you want to know your epigenetic age:**

Counts/frequency:
Not at all (1, 5.9%)
Not much (0, 0.0%)
Neutral (1, 5.9%)
Somewhat (2, 11.8%)
Very much (13, 76.5)

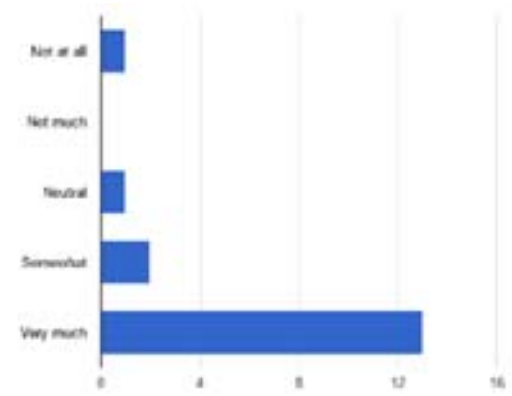

2. For question #1, why did you select that answer?

- "I want to know about my health"
- "I am curious to know my epigenetic age"
- "I do not want to know"
- "I always want to know how my body is responding to age."
- "I feel the more information I have about my health the better informed choices I can make about lifestyle, medical decisions, etc."
- "It's a field of study I'm very interested to learn more about."
- "It would be easier if one were to know their epigenetic age to help understand your own body as you age."
- "It may improve choices in the future if I can slow down the cell aging process."
- "I am neutral about knowing because I'm not a medical expert."
- "I want to see my actual age."

3. Before beginning this study, if you were told your epigenetic age appeared more advanced than your chronological age, do you think you would be more or less motivated to eat the nuts and olive oil everyday

Counts/frequency:
Not motivated at all (0, 0.0%)
Less motivated (1, 5.9%)
Neutral (2, 11.8%)
More motivated (5, 29.4%)
Very motivated (9, 52.9%)

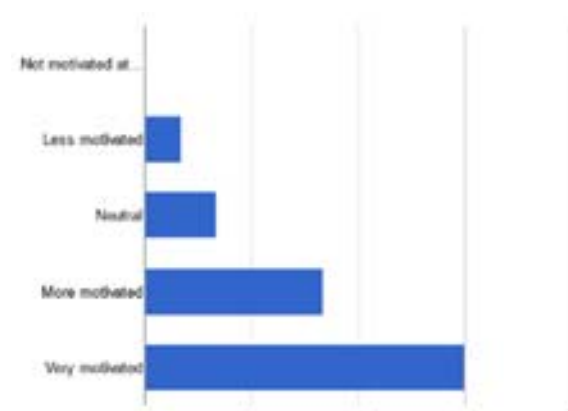

4. Before beginning this study, if you were told your epigenetic age appeared younger than your chronological age, do you think you would be more or less motivated to eat the nuts and olive oil everyday?

Counts/frequency:
Not motivated at all (0, 0.0%)
Less motivated (0, 0.0%)
Neutral (6, 35.3%)
More motivated (4, 23.5%)
Very motivated (7, 41.2%)

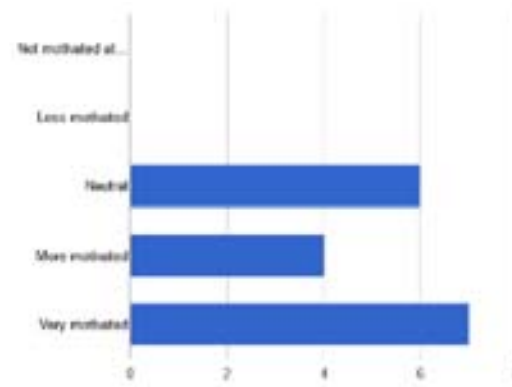

5. If eating tree nuts and olive oil slowed biological aging, how likely would you be to continue to eat tree nuts and olive oil daily?

Counts/frequency:
Very unlikely (0, 0.0%)
Unlikely (0, 0.0%)
Neutral (1, 14.3%)
Somewhat likely (2, 28.6%)
Very likely (4, 57.1%)

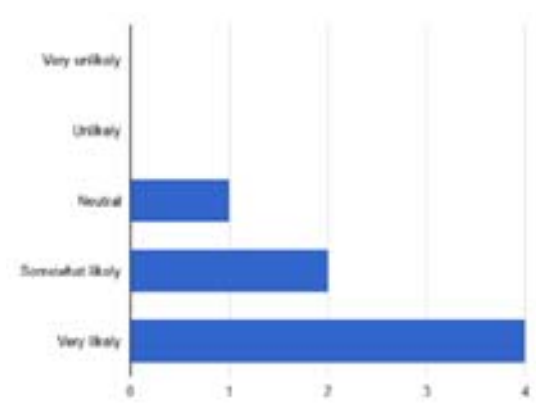

(Version 2 Question) 5. If eating tree nuts slowed biological aging, how likely would you be to continue to eat tree nuts daily?

Counts/frequency:
Very unlikely (1, 9.1%)
Unlikely (0, 0.0%)
Neutral (0, 0.0%)
Somewhat likely (1, 9.1%)
Very likely (9, 81.8%)

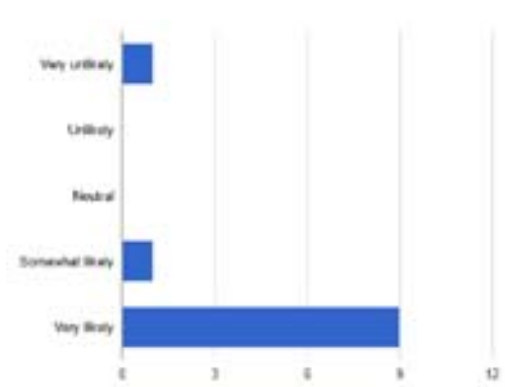

(Version 2 Question) 6. If consuming olive oil slowed biological aging, how likely would you be to continue to consume olive oil daily?

Counts/frequency:
Very unlikely (1, 9.1%)
Unlikely (0, 0.0%)
Neutral (0, 0.0%)
Somewhat likely (1, 9.1%)
Very likely (9, 81.8%)

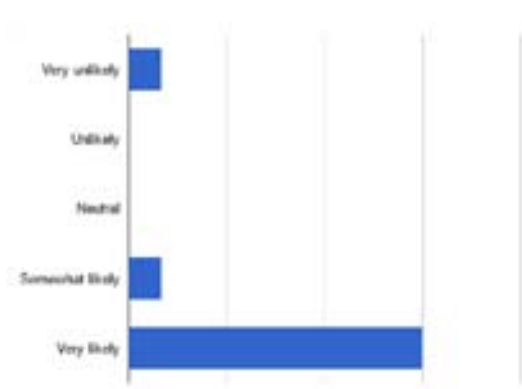

(7. Version 2) 6. Do you feel we did a good job explaining the concept of epigenetic aging?

Counts/frequency:
Not at all (0, 0.0%)
Not much (0, 0.0%)
Neutral (1, 5.9%)
Somewhat (3, 17.6%)
Very much (13, 76.5%)

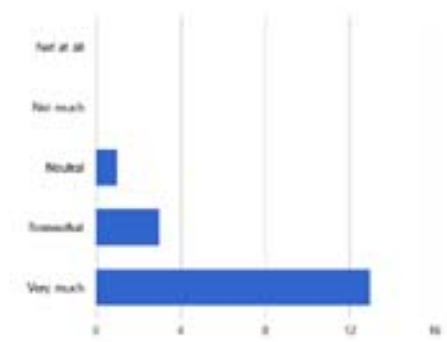

(8. Version 2) 7. For question #6, can you comment on why you selected that answer?

- "Considering I have never heard of epigenetic aging. Each conversation with the staff gave me a better understanding."
- "Explained very well"
- "It's a new concept for me"
- "I had no idea what this concept was before this study. I still don't completely understand but I feel I have a general idea and concept."
- "I participated in several other studies and this one was one of the better ones."
- "I would have liked more information."
- "If it is healthy I like it"
- "It was explained in detail. Prior to this study I had no clue about epigenetic aging. I also reviewed the handout."
- "It was explained to me clearly"
- "Not much discussion on this subject. Only a flyer that described what it was."
- "Your explanation was in writing which gave me the opportunity to read it twice and absorb the information"
